# Association of Dietary Magnesium Intake With Leukocyte Telomere Length in United States Middle-Aged and Elderly Adults

**DOI:** 10.3389/fnut.2022.840804

**Published:** 2022-05-19

**Authors:** Lihua Hu, Yi Bai, Guiping Hu, Yan Zhang, Xiaoning Han, Jianping Li

**Affiliations:** ^1^Department of Cardiology, Peking University First Hospital, Beijing, China; ^2^Department of Epidemiology and Bio-Statistics, School of Public Health, Peking University, Beijing, China; ^3^School of Engineering Medicine, Beihang University, Beijing, China; ^4^Beijing Advanced Innovation Center for Big Data-Based Precision Medicine, Advanced Innovation Centre for Biomedical Engineering, Beihang University, Beijing, China

**Keywords:** leukocyte telomere length, nutrition, dietary magnesium intake, lifespan, National Health and Nutrition Examination Survey

## Abstract

**Aim:**

Magnesium supplementation may extend the life span; however, the biological mechanism is still unknown. Leukocyte telomere length (LTL) is a marker of cell aging and biological health in humans. Data concerning whether magnesium supplementation can maintain telomere length, thus prolonging life are limited. We aimed to investigate the association between dietary magnesium intake and LTL in United States middle-aged and elderly adults.

**Methods:**

A total of 4,039 United States adults aged ≥ 45 years from National Health and Nutrition Examination Survey (1999–2002). Dietary magnesium intake was collected by a trained interviewer using 24-h dietary recall method and LTL was obtained using the quantitative polymerase chain reaction method. Multiple linear regression analysis was performed to evaluate the crude and adjusted association of dietary magnesium intake with LTL.

**Results:**

The overall mean (SD) of LTL was 5.6 (0.6) kp. After adjusting potential confounders, every 1 mg increase in log-transformed dietary magnesium intake was associated with 0.20 kp (95% confidence intervals: 0.05–0.34) longer LTL. Participants with the highest tertile (≥299 mg) of dietary magnesium intake had statistically significant longer LTL (β = 0.07, *P* = 0.038) compared with the lowest tertile (<198 mg), with significant linear trends across tertiles. Moreover, the association between dietary magnesium intake and LTL was significantly stronger in participants with higher levels of education (≥high school compared with < high school, *P* for interaction = 0.002). *E*-value analysis suggested robustness to unmeasured confounding.

**Conclusion:**

Our findings showed that increased dietary magnesium intake was associated with longer LTL, which suggested that magnesium was conducive to a longer life expectancy.

## Introduction

Telomeres, the TTAGGG repetitive DNA at the ends of linear chromosomes, are important and active controllers of cellular lifespan and chromosome integrity in eukaryotes cells (1). Telomere attrition is an integral part of the end replication problem (2); thus, leukocyte telomere length (LTL) shortening has been viewed as a useful bioindicator for cellular aging (3). In addition, acceleration of the rate of telomeric sequence loss is a feature of a plethora of adverse health outcomes (4, 5). Shortened LTL been reported to be linked with increased risk for numerous chronic conditions, including cardiovascular disease (CVD; 6–8), diabetes mellitus (9, 10), Alzheimer’s disease (11), hypertension (12), and cancer (13). Growing evidence suggests that LTL can be influenced by lifestyle factors, such as smoking, physical activity and energy intake (14–16). Recently, the importance of nutritional factors on LTL has been increasingly recognized (17).

Magnesium is an essential element, as a cofactor, by over 300 enzymatic reactions required to maintain homeostasis (18). Diet is the major source of magnesium in human. Nuts, seeds, leafy vegetables, or whole-grain cereals are well-recognized dietary sources of magnesium (19). Unbalanced magnesium intake can cause adverse health effects (19, 20). Observational studies have shown that magnesium deficiency is associated with poor cardio-metabolic conditions (20). An increasing body of epidemiologic evidence reported that higher dietary magnesium intake could exert beneficial effects on CVD risk factors by improving glucose and insulin metabolism, ameliorating lipid profile and by its actions as an antihypertensive and anti-inflammatory agent (19). Previous studies have shown that magnesium intake may extend the life span. The process is considered to be associated with the involvement of magnesium in many metabolic processes including ATP-dependent biochemical reactions, synthesis of DNA, RNA expression, cellular excitability, and cellular health span (21). However, whether LTL plays a role in prolonging lifespan with magnesium intake remains unclear. Although some studies have explored the associations between minerals intake (e.g., copper, zinc, and selenium) and LTL (22–24), the association of dietary magnesium intake with LTL has rarely been examined. Of note, the possible effect modifiers for the dietary magnesium intake-LTL association have not been fully investigated in previous studies. Therefore, the present study aimed to address the knowledge gap by examining the association of dietary magnesium intake with LTL and to explore any possible effect modifiers in United States middle-aged and elderly adults using a large population-based survey data, the National Health and Nutrition Examination Survey (NHANES).

## Materials and Methods

### Study Design and Population

National Health and Nutrition Examination Survey, conducted by the Centers for Disease Control and Prevention, was an ongoing repeated cross-sectional study designed to assess the health and nutritional status of adults and children in the United States. The Ethics Review Board of the National Center for Health Statistics approved the NHANES study protocols. Written informed consents were obtained from all study participants. More detailed information is available at http://www.cdc.gov/nchs/nhanes.htm.

**FIGURE 1:**
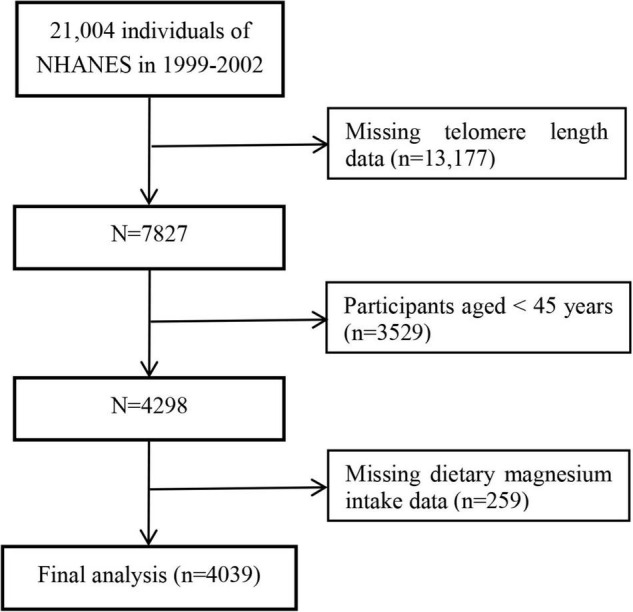
Flow chart of the study participants.

The data from NHANES 1999–2000 and 2001–2002 were combined for these analyses because LTL was assessed in these two data collection cycles. In total, 7,827 participants with LTL data were enrolled. Considering that LTL was associated with age-related chronic diseases and middle-aged and elderly people were more prone to malnutrition, we excluded participants aged < 45 years old (*n* = 3,529) and with missing information on dietary magnesium intake (*n* = 259). Finally, a total of 4,039 participants were included in this study (Figure 1).

### Dietary Magnesium Intake

Dietary intake information was assessed *via* 24 h recall obtained by a trained interview from what we eat in America survey which was conducted in the Mobile Examination Center. The types and amounts of all foods and beverages during the 24-h period prior to the interview were collected with the use of a computer-assisted dietary interview system. The dietary magnesium intake was estimated based on the University of Texas Food Intake Analysis System and United States Department of Agriculture Survey Nutrients Database (22). The nutrient estimates did not include nutrients obtained from medications or dietary supplements.

### Leukocyte Telomere Length Assessment

The detailed methods for LTL quantification have been described previously (12, 23, 25). The telomere length relative to standard reference DNA (T/S ratio) was obtained in the blood leukocytes using real-time quantitative polymerase chain reaction (26). Each sample was assigned to duplicate wells in a 96 well plate and assayed three times on three different days. Each panel contained eight control DNA samples to normalize the variability between the two tests. The inter-assay coefficient of variation was 6.5%. The formula of the conversion from T/S ratio to kp is [3,274 + 2,413 × (T/S)]/1,000 (25). The conversion from T/S ratio to kp was calculated based on a comparison of telomeric restriction fragment length from Southern blot analysis and T/S ratios using DNA samples from the human diploid fibroblast cell line IMR90 at different population doublings.

### Covariates

The covariates were selected based on previous studies examining risk factors for LTL as well as adjusting for covariates that, when added to this model, changed the matched odds ratio by at least 10%. The following variables were used to construct the fully adjusted model. Continuous variables included age (years), body mass index (BMI, kg/m^2^), poverty to income ratio (PIR), sample weight, alcohol consumption (gm), total cholesterol (TC, mg/dL), high density lipoprotein cholesterol (HDL-C, mg/dL), triglycerides (TG, mg/dL), fasting blood glucose (FBG, mg/dL), dietary fiber (g), and total energy intake (kcal). Categorical variables consisted of sex (male, female), race/ethnicity (non-Hispanic white, non-Hispanic black, Mexican American, other Hispanic, or other), education (less than high school, high school, or greater than high school), smoking (never smoker, former smoker, or current smoker), physical activity (sedentary, low, moderate, or high), hypertension, and self-reported history of diabetes. Hypertension was defined as having a history of hypertension, a systolic blood pressure ≥ 140 mmHg and/or diastolic blood pressure ≥ 90 mmHg, and using antihypertensive medications (12, 27).

### Statistical Analysis and Sensitivity Analysis

Sample weights were used for analyses to account for the complex survey design and non-response of NHANES (28). Weighted means, proportions and standard error (Se) were calculated for baseline characteristics using survey sample weights. A weighted linear regression model (continuous variables) or weighted chi-square test (categorical variables) were used to calculate differences among different dietary magnesium intake groups (tertiles). Because the distribution of values for dietary magnesium intake was strongly skewed toward the upper end, the dietary magnesium intake was log-transformed to better approximate a normal distribution. We applied multiple linear regression analysis to evaluate the independent association between dietary magnesium intake and LTL. We constructed two adjusted models: Model 1 was adjusted for age, sex, race, education status, smoking status, alcohol consumption, and PIR; model 2 was further adjusted for physical activity, BMI, hypertension, diabetes, TC, TG, HDL-C, FBG, sample weight, dietary fiber, and total energy intake. Results were presented in coefficients (β) with the corresponding 95% confidence intervals (CIs). Multiple linear analysis with cubic spline functions model and smooth curve fitting (penalized spline method) was further conducted to characterize the shape of the relationship between dietary magnesium intake and LTL (29).

To ensure the robustness of the data analysis, we also did sensitivity analyses. We performed testing for linear trends by entering the median value of each category of dietary magnesium intake as a continuous variable in the models. Moreover, we explored the potential for unmeasured confounding between dietary magnesium intake and LTL by calculating *E*-values. The *E*-value quantifies the required magnitude of an unmeasured confounder that could negate the observed association between dietary magnesium intake and LTL. In addition, possible modifications of the association between dietary magnesium intake and LTL were also assessed for the following variables: sex (females vs. males), age (<65 vs. ≥65 years), BMI (<30 vs. ≥30 kg/m^2^), current smoking (yes vs. no), education (<high school vs. ≥high school), diabetes (yes vs. no), and hypertension (yes vs. no).

All *P* values were two-sided with a significance level of <0.05. The analyses were performed using the statistical package R^1^.

## Results

### Baseline Characteristics of Study Participants

**FIGURE 2:**
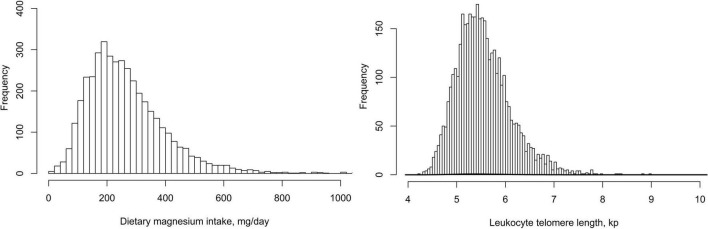
The distributions of dietary magnesium intake and leukocyte telomere length.

A total of 4,039 study participants aged 45–85 years [weighted mean age: 15.5 (2.3) years; 47.4% men] were included in this final data analysis. As shown in Figure 2, the mean (Se) magnesium intake was 270.7 (134.8) mg (median 246.0 mg). The mean (Se) LTL was 5.6 (0.6) kp (median 5.5 kp). The distributions of dietary magnesium intake and LTL were skewed toward the upper end. The weighted distributions of 4,039 participants’ sociodemographic characteristics and other covariates according to dietary magnesium intake tertiles were shown in Table 1. The ranges of dietary magnesium intake for tertiles 1–3 were <198, 198–299, and ≥299 mg, respectively. Compared with tertile 1 and tertile 2 of dietary magnesium intake, participants in tertile 3 seemed to be younger, to be more males and non-Hispanic whites, to have higher educational levels and physical activity levels, to have a lower rate of current smoking and hypertension, to have higher values in PIR, alcohol intake, dietary fiber, total energy intake and LTL and to have lower values in BMI and HDL-C (all *P* < 0.01).

**TABLE 1:** Weighted characteristics of study population based on tertiles of dietary magnesium intake.

Variables*	Total participants	Dietary magnesium intake, mg			*P* value^¶^
		Tertile 1 (<198)	Tertile 2 (198–299)	Tertile 3 (≥299)	
*N* ^†^	4,039	1,346	1,346	1,347	
Male,%	47.4	30.7	42.0	65.7	<0.001
Age, years	60.1 (11.4)	62.2 (11.9)	60.0 (11.6)	58.6 (10.6)	<0.001
BMI, kg/m^2‡^	28.6 (6.2)	28.9 (6.3)	28.7 (6.1)	28.1 (6.1)	0.004
PIR	3.2 (1.6)	2.8 (1.6)	3.3 (1.6)	3.5 (1.6)	<0.001
Race,%					<0.001
Non-Hispanic white	80.2	74.4	80.2	84.7	
Non-Hispanic black	7.3	12.2	6.8	3.9	
Mexican American	3.8	4.2	3.5	3.8	
Other Hispanic	5.6	5.6	5.7	5.5	
Other race	3.1	3.6	3.8	2.1	
Education,%					<0.001
<High school	22.8	33.1	21.7	15.5	
High school	25.6	28.4	25.7	23.1	
>High school	51.7	38.5	52.6	61.4	
Smoking,%					<0.001
Never	45.6	48.0	46.9	42.5	
Former	35.8	27.9	35.1	42.6	
Current	18.6	24.1	18.0	14.9	
Alcohol, gm/day	9.3 (27.6)	5.0 (18.8)	8.3 (22.8)	13.8 (35.7)	<0.001
Physical Activity,%^¶¶^					<0.001
Sedentary	24.7	31.9	25.0	18.4	
Low	27.2	29.8	23.5	28.5	
Moderate	18.0	14.8	20.4	18.4	
High	30.1	23.5	31.1	34.6	
Hypertension,%	41.9	46.9	41.3	38.4	<0.001
Diabetes,%	13.6	15	12.8	13.3	0.251
FBG, mg/dL	100.7 (36.6)	101.4 (39.5)	100.1 (34.6)	100.6 (35.9)	0.652
TC, mg/dL	213.4 (41.6)	214.9 (40.4)	213.4 (39.3)	212.1 (44.6)	0.221
TG, mg/dL	158.9 (140.5)	158.5 (113.7)	158.5 (165.4)	159.5 (135.2)	0.974
HDL-C, mg/dL	52.5 (16.2)	52.8 (16.4)	53.7 (16.5)	51.1 (15.5)	<0.001
Total energy intake, kcal	2,008.5 (867.3)	1,344.9 (477.3)	1,914.0 (595.4)	2,624.0 (892.3)	<0.001
Fiber intake, *g*	16.2 (10.0)	8.8 (4.0)	14.3 (5.2)	23.9 (11.2)	<0.001
Magnesium intake, mg	270.7 (134.8)	150.8 (31.9)	247.3 (29.1)	416.0 (118.1)	<0.001
Telomere length, T/S ratio	1.0 (0.2)	0.9 (0.2)	1.0 (0.2)	1.0 (0.2)	<0.001
LTL, kp	5.6 (0.6)	5.5 (0.6)	5.6 (0.6)	5.6 (0.6)	<0.001

*BMI, body mass index; PIR, family poverty income ratio; FBG, fasting blood glucose; TC, total cholesterol; TG, triglycerides; HDL-C, high density lipoprotein cholesterol; and LTL, leukocyte telomere length.*

*Mean and standard error (Se) for continuous variables and percentages for categorical variables were weighted. ^¶^P values of continuous variables and categorical variables were calculated by weighted linear regression model and weighted chi-square test, respectively. ^†^Unweighted sample number in the dataset.

*^‡^BMI was calculated as the body weight in kilograms divided by the square of the height in meters.*

*^¶¶^ The physical activity categories were based on the distribution of MET-minute levels for the present NHANES sample.*

### Association Between Dietary Magnesium Intake and Leukocyte Telomere Length

**FIGURE 3:**
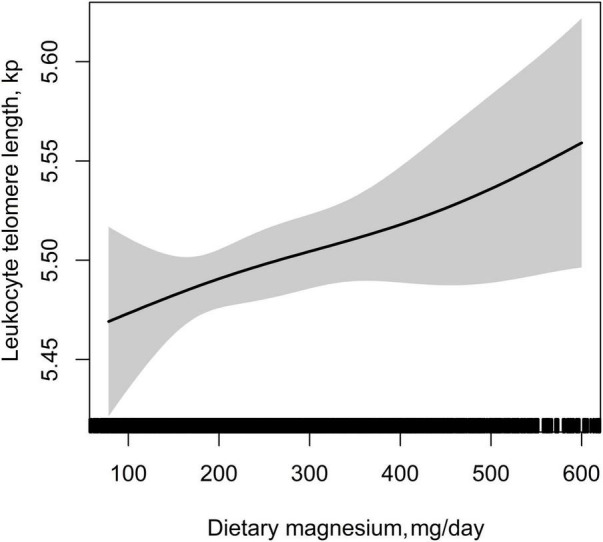
Dose–response relationship between dietary magnesium intake and leukocyte telomere length. Adjusted for age, sex, BMI, race, education status, smoking status, alcohol consumption, physical activity, PIR, hypertension, diabetes, TC, TG, HDL-C, FBG, sample weight, dietary fiber, and total energy intake.

Table 2 showed the association between dietary magnesium intake and LTL using multiple linear regression analyses. In the crude model, log-transformed dietary magnesium intake, as a continuous variable, was positively associated with longer LTL (β = 0.11, 95% CI: 0.03–0.20, *P* = 0.009). The association between dietary magnesium intake and LTL was still observed after adjustment for different potential confounders (all *P* < 0.01). In the fully adjusted model (Model II), every 1 mg increase in log-transformed dietary magnesium intake was associated with 0.20 kp (95%CI: 0.05–0.34) longer LTL. We also converted dietary magnesium intake from a continuous variable to a categorical variable (tertiles). Compared with the tertile 1 of dietary magnesium intake (<198 mg), the participants in the highest tertile (dietary magnesium intake ≥299 mg) had statistically significant longer LTL (β = 0.07, 95% CI: 0.01–0.13). Tests for linear trend (*P* for trend) were significant and consistent with the *P*-value when dietary magnesium intake was used as a continuous variable, suggesting a significant linear trend among tertiles of dietary magnesium intake and LTL. We further explored the shape of the dose-response relationship by using GAM models. As shown in Figure 3, the association of dietary magnesium intake with LTL was linear.

**TABLE 2:** Association of dietary magnesium intake with leukocyte telomere length among middle-aged and elderly population (*n* = 4,039).

Dietary magnesium intake, mg	Leukocyte telomere length, kp					
	Crude model		Model I		Model II	
	β (95% CI)	*P* value	β (95% CI)	*P* value	β (95% CI)	*P* value
Continuous^†^	0.11 (0.03, 0.20)	0.009	0.18 (0.03, 0.32)	0.016	0.20 (0.05, 0.34)	0.009
**Tertiles**						
Tertile 1 (<198)	0 (Reference)		0 (Reference)		0 (Reference)	
Tertile 2 (198–299)	0.05 (0.01, 0.09)	0.028	0.05 (0.00, 0.10)	0.056	0.05 (0.01, 0.10)	0.037
Tertile 3 (≥299)	0.05 (0.01, 0.09)	0.018	0.06 (0.01, 0.13)	0.047	0.07 (0.01, 0.13)	0.038
*P* for trend	0.018		0.047		0.039	

*CI, confidence interval. ^†^Dietary magnesium intake value was log-transformed.*

*Model I was adjusted for age, sex, race, education status, smoking status, alcohol consumption, PIR, sample weight, physical activity, dietary fiber, and total energy intake. Model II was further adjusted for BMI, hypertension, diabetes, TC, TG, HDL-C, and FBG.*

### Sensitivity Analysis

We generated an *E*-value to assess the sensitivity to unmeasured confounding. Dietary magnesium intake was positively associated with an increase in LTL by multivariable analysis (β = 0.20, 95% CI: 0.05–0.34). The *E*-value was 2.05, meaning that residual confounding could explain the observed association if there exists an unmeasured covariate having a relative risk association ≥2.05 with both dietary magnesium intake and LTL. Therefore, it is unlikely that an unmeasured or unknown confounder would have a substantially greater effect on LTL than these known risk factors. Consistently, the primary findings were robust.

**FIGURE 4:**
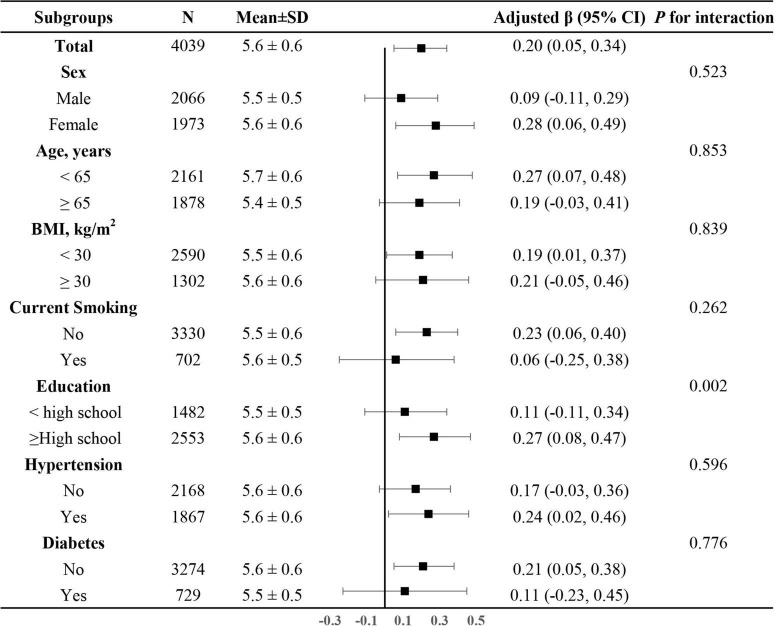
The association between log-transformed dietary magnesium intake on leukocyte telomere length in various subgroups. Adjusted for age, sex, BMI, race, education status, smoking status, alcohol consumption, physical activity, PIR, hypertension, diabetes, TC, TG, HDL-C, FBG and sample weight, dietary fiber, and total energy intake, if not be stratified.

In addition, we further explored the role of covariables in the association between dietary magnesium intake and LTL. As shown in Figure 4, the effect of log-transformed dietary magnesium intake on LTL was significantly stronger in participants with higher levels of education (<high school: β = 0.11, 95%CI: −0.11–0.34; ≥high school: β = 0.27, 95% CI: 0.08–0.47, *P* for interaction = 0.002). None of the other variables, including sex, age, BMI, current smoking, diabetes, and hypertension, showed significant effect modification on the association between dietary magnesium intake and LTL (all *P* for interaction > 0.05).

## Discussion

In this study, dietary magnesium intake was independently and positively associated with longer LTL in United States middle-aged and elderly participants, even after adjusting for covariates. Furthermore, this association was more pronounced in populations with higher levels of education. Our findings suggest that magnesium supplementation may extend the life span. Further research is needed to determine what would be the optimal range of dietary magnesium intake for the Chinese population.

We focused on dietary magnesium and its association with LTL because a suggestion of magnesium dietary supplement for Chinese adults is unknown. Prior reviews indicated that magnesium might have protective effects on telomere attrition (30) and correcting magnesium deficiencies might prolong life (31). However, whether LTL plays a role in prolonging lifespan with magnesium intake remains unclear. Of note, only a few previous studies have reported the association between magnesium and LTL with inconsistent results. Mazidi et al. (24) used data from NHAENS 1999–2001 and found mean (adjusted for sex, age, and race) dietary intakes of magnesium monotonically increased across TL quarters. Yu et al. (32) conducted a cross-sectional study of 467 participants with a mean magnesium intake of 247.13 mg/day and found an inverse relationship between dietary magnesium and LTL. O’Callaghan et al. (33) used data from 23 South Australians aged 65 years or older and found a negative association between telomere length and serum magnesium levels (*r* = −0.61, *P* = 0.001, respectively). The previous studies have many limitations, such as a small sample size and simple statistical methods. They had thus far only focused on exploring the correlation, not multiple regression analysis. Besides, none of them discussed the possibility of a non-linear relationship. Furthermore, whether this association was modified by some risk factors of LTL needs to be verified. Magnesium is the second most abundant cation in cellular systems and plays a role in numerous biological functions including the maintenance of DNA structure, thus it is difficult to reconcile the role of high magnesium in telomere shortening.

In our study, we provided some new insights into this field. First, we observed that dietary magnesium intake was positively associated with longer LTL. Also, our study confirmed a significant linear relationship between dietary magnesium intake and LTL using smooth curve fitting. The findings suggested that magnesium was conducive to longer life expectancy which might have an intimate connection with LTL. Several possible mechanisms for this association include (1) Magnesium plays a role in telomere maintenance and the activity of telomerase; thus, increasing magnesium supplement could extend LTL (31). (2) Long-term intake of magnesium has demonstrated anti-inflammatory and antioxidative properties which may impact LTL (34, 35). However, more studies are needed to confirm our results and further examine the underlying mechanisms. Second, subgroup analyses showed that education level was a significant modifier: a stronger association was found in participants with higher levels of education (≥high school). The biological mechanism underlying high levels of education × high dietary magnesium intake is still unknown. In our current study, participants with higher levels of education had longer LTL; however, among those with lower education levels, the LTL was shortened. Some studies reported that excess sedentary behavior and lack of physical activity could contribute to shortened telomeres (36, 37). A plausible biological explanation for the interaction may be since poor lifestyle, behavior or environmental conditions related to lower education level has an indirect influence on the association between dietary magnesium intake and LTL. Moreover, one study demonstrated that low educational attainment might be an indicator of long-term socioeconomic status trajectories, and be associated with accumulated allostatic load resulting in telomere shortening (38). Taken together, further research is needed to examine the association between education level, dietary magnesium intake and LTL.

The mean level of dietary magnesium intake as seen in our study was 270.7 mg/day, which was less than the general recommendations for 300 mg magnesium intake a day. Although serum magnesium was also unavailable, the data from NHANES 2006 showed that low serum magnesium concentrations were 36.3 and 31.0% for females and males, respectively, (39). Moreover, epidemiological studies reported that dietary magnesium intake was inadequate in the United States population as well as in other populations (40, 41). 68% of Americans (40) and 72% of middle-aged French adults have been shown to consume below the recommended intake of magnesium. These findings suggested that dietary magnesium intake was inadequate in United States populations. Thus, increasing the dietary magnesium intake in the United States population may be an important public health goal.

Our study findings have important clinical implications. Data from the present study show a positive association between dietary magnesium intake and LTL in United States middle-aged and elderly participants. Hence, our findings suggest that magnesium supplementation may extend the life span. If further confirmed, our findings may provide important data for clinical and nutritional guidelines on optimal magnesium intake. Furthermore, one future direction of this work is to clarify the potential mechanisms of educational disparities in dietary magnesium and LTL.

Our study has some strengths. First, this study was the first report to explore the association between dietary magnesium intake and LTL in United States middle-aged and elderly adults. Second, the study populations were randomly invited to NHANES which applied rigorous quality controls to the procedures. Third, we provided an adequate statistical rationale to evaluate the association between dietary magnesium intake and LTL, a feature that was lacking in previous studies. Fourth, the exact shape of the dose-response relationship between dietary magnesium intake and LTL was the first report using smooth curve fitting. Some limitations of our study should be also noted. First, as a cross-sectional design, it had less power to infer the causal association between dietary magnesium intake and LTL. Further prospective cohort studies are needed to verify these findings. Second, as in all observational studies, even though known potential confounding factors were controlled for, there might have been still uncontrolled confounding due to unmeasured differences in behaviors or other dietary habits. We used the *E*-value sensitivity analysis to quantify the potential implications of unmeasured confounders and found that an unmeasured confounder was unlikely to explain the entirety of the dietary magnesium intake effect. Third, fruit, vegetable, and whole-grain intake were not adjusted. However, the dietary magnesium intake was estimated based on the types and amounts of all foods and beverages. Fourth, the dietary intake was assessed by 24-h dietary recall interviews which might lead to imprecise estimates due to day-to-day variations in diet. Additionally, it is well known that LTL shows seasonal variation. The availability for the season/month of blood draw was not adjusted because NHANES did not provide data.

## Conclusion

In summary, this study demonstrated that dietary magnesium intake was independently and positively associated with LTL in United States middle-aged and elderly participants, and this association was more pronounced in populations with higher levels of education. Moreover, the findings underscore the need to further research to establish optimal range of magnesium dietary supplements in the Chinese population. By doing so, it would provide more specific clinical and nutritional guidelines on optimal magnesium intake.

## Data Availability Statement

The datasets presented in this study can be found in online repositories. The names of the repository/repositories and accession number(s) can be found below: The datasets are available on DataDryad (https://doi.org/10.5061/dryad.d5h62).

## Ethics Statement

The studies involving human participants were reviewed and approved by the Ethics Review Board of the National Center for Health Statistics (NCHS) and approved the NHANES study protocols. The participants provided their written informed consent to participate in this study.

## Author Contributions

LH, GH, and JL conceived and designed the research, and were involved in funding acquisition. LH, YB, and GH participated in acquisition of data, or analysis and interpretation of data. LH wrote the original draft manuscript. GH, YZ, XH, and JL reviewed and edited the manuscript. All authors approved the final version of the manuscript and agreed to be accountable for all aspects of the work.

## Conflict of Interest

The authors declare that the research was conducted in the absence of any commercial or financial relationships that could be construed as a potential conflict of interest.

## Publisher’s Note

All claims expressed in this article are solely those of the authors and do not necessarily represent those of their affiliated organizations, or those of the publisher, the editors and the reviewers. Any product that may be evaluated in this article, or claim that may be made by its manufacturer, is not guaranteed or endorsed by the publisher.

## Footnotes

1 http://www.R-project.org, The R Foundation.

## Abbreviations

LTL: leukocyte telomere length
NHANES: National Health and Nutrition Examination Survey
PCR: polymerase chain reaction
CI: confidence intervals
CVD: cardiovascular disease
BMI: body mass index
PIR: family poverty income ratio
FBG: fasting blood glucose
TC: total cholesterol
TG: triglycerides
HDL-C: high density lipoprotein cholesterol.
